# Implementation of a Novel Method for Processing Proteins from Acetic Acid Bacteria via Liquid Chromatography Coupled with Tandem Mass Spectrometry

**DOI:** 10.3390/molecules29112548

**Published:** 2024-05-29

**Authors:** Juan J. Román-Camacho, Juan C. Mauricio, Irene Sánchez-León, Inés M. Santos-Dueñas, Carlos A. Fuentes-Almagro, Francisco Amil-Ruiz, Teresa García-Martínez, Isidoro García-García

**Affiliations:** 1 Department of Agricultural Chemistry, Edaphology and Microbiology Agrifood Campus of International Excellence ceiA3, University of Cordoba, 14014 Cordoba, Spain; b32rocaj@uco.es (J.J.R.-C.); b32salei@uco.es (I.S.-L.); mi2gamam@uco.es (T.G.-M.); 2 Department of Inorganic Chemistry and Chemical Engineering, Agrifood Campus of International Excellence ceiA3, Institute of Chemistry for Energy and Environment (IQUEMA), University of Cordoba, 14014 Cordoba, Spain; ines.santos@uco.es (I.M.S.-D.); isidoro.garcia@uco.es (I.G.-G.); 3 Proteomics Unit, Central Service for Research Support (SCAI), University of Cordoba, 14014 Cordoba, Spain; proteomica2@uco.es; 4 Bioinformatics Unit, Central Service for Research Support (SCAI), University of Cordoba, 14014 Cordoba, Spain; bioinformatica@uco.es

**Keywords:** acetic acid bacteria, LC–MS/MS, proteins, vinegar

## Abstract

Acetic acid bacteria (AAB) and other members of the complex microbiotas, whose activity is essential for vinegar production, display biodiversity and richness that is difficult to study in depth due to their highly selective culture conditions. In recent years, liquid chromatography coupled with tandem mass spectrometry (LC–MS/MS) has emerged as a powerful tool for rapidly identifying thousands of proteins present in microbial communities, offering broader precision and coverage. In this work, a novel method based on LC–MS/MS was established and developed from previous studies. This methodology was tested in three studies, enabling the characterization of three submerged acetification profiles using innovative raw materials (synthetic alcohol medium, fine wine, and craft beer) while working in a semicontinuous mode. The biodiversity of existing microorganisms was clarified, and both the predominant taxa (*Komagataeibacter*, *Acetobacter*, *Gluconacetobacter*, and *Gluconobacter*) and others never detected in these media (*Asaia* and *Bombella*, among others) were identified. The key functions and adaptive metabolic strategies were determined using comparative studies, mainly those related to cellular material biosynthesis, energy-associated pathways, and cellular detoxification processes. This study provides the groundwork for a highly reliable and reproducible method for the characterization of microbial profiles in the vinegar industry.

## 1. Introduction

According to numerous ecological and phylogenic studies, acetic acid bacteria (AAB) belong to a distinctive taxonomic group within the Acetobacteraceae family [1,2]. AAB stand out as strict aerobic microorganisms that are Gram-negative or Gram-variable and catalase-positive and oxidase-negative, which present an optimum growing temperature between 25 and 30 °C and grow in a wide range of considerably low pH between 2.5 and 6.5 [3,4]. Moreover, these microorganisms may be found in a wide variety of natural and industrial environments, such as those foodstuffs whose main component is acetic acid, especially vinegar [5].

Regarding their metabolism, AAB are traditionally known for their ability to incompletely oxidize various carbon sources, mainly alcohols, into organic acids. The biotransformation of ethanol into acetic acid, catalyzed by the enzymes pyrroloquinoline quinone-dependent alcohol dehydrogenase (PQQ-ADH) and membrane-bound aldehyde dehydrogenase (ALDH), is the key pathway for vinegar production [6,7,8]. Vinegar is likely one of the most appreciated products in the fermented food industry and one of the most economically important products obtained from AAB [5,9]. For this reason, among others, much of the research on the metabolism of AAB is aimed at improving the procedures for obtaining this product [10,11,12]. Because diverse AAB species may also metabolize sugars and sugar alcohols, the role of membrane-bound soluble dehydrogenases is attracting increasing interest from researchers. This interest stems from AAB species’ capability to carry out the incomplete oxidation of different substrates in the development of innovative processes [8,13,14]. However, despite the importance of this oxidative metabolism, which is crucial for vinegar production, studies on other metabolic pathways, including the biosynthesis of cellular materials, energy metabolism, and membrane detoxification mechanisms, which are part of the molecular strategies used by the microbiota for survival in these aggressive media, are also of great interest [15,16,17,18,19].

In recent years, it has been acknowledged that vinegar is a product obtained not only by the activity of AAB but also as the result of complex microbiota activity in which other members of the Acetobacteraceae family, as well as other bacteria groups such as lactic acid bacteria and even archaea, also participate [20,21,22]. Despite this, numerous essential aspects of the molecular mechanisms driving the overall activity of these microorganisms are not yet comprehensively well-known, despite their significance in the development of these bioprocesses. This fact may be because the AAB and other microorganisms commonly found in the industry, especially in submerged cultures developed in bioreactors, exhibit highly selective growing conditions, including high ethanol-oxidizing ability, acetic acid requirement, tolerance to high acidity levels [10–20% (*w*/*v*)], low pH, and a constantly demand for oxygen throughout the process [2,5,9]. For this reason, the isolation of members of these communities outside the media where they usually work is still, to date, a challenge for researchers attempting to obtain a selection of starter cultures for the industry, as well as to improve the knowledge of the richness of these complex media [23].

Omics tools are starting to be applied in the AAB sector with the aim of overcoming the disadvantages of traditional methods in the study of the diversity of these microorganisms without the need for isolation [24,25,26,27]. Among the different omics areas, proteomics and metaproteomics offer numerous advantages, such as the possibility of identifying different microorganisms from their proteins, functional studies, more precise protein quantification, and analysis of protein–protein interactions, among others [28]. Since the early days in the field of proteomics using AAB, the first gel-based electrophoretic techniques, such as two-dimensional electrophoresis (2DE), differential gel electrophoresis (DIGE), and 2D-DIGE, allowed the identification of proteins in response to acid stress [25,29,30]. Given the laborious nature of these electrophoretic methods, the development of new approaches for the rapid and routine identification of bacteria was driven by the implementation of matrix-assisted laser desorption ionization time-of-flight mass spectrometry (MALDI-TOF MS) [31]. This technique allows the rapid identification of AAB involved in industrial vinegar production. More recently, the merger of liquid chromatography with mass spectrometry (MS) technology gave rise to liquid chromatography coupled with tandem mass spectrometry (LC–MS/MS) [32,33]. This analytic tool highlights the protein-based techniques applied to the omics area because it provides a wide dynamic range and protein coverage without the need for isolation, making it advantageous for studies in situ by providing detailed insights into microbiota composition and behavior working throughout a bioprocess, as is the case of vinegar elaboration [29,32,34]. However, LC–MS/MS also presents limitations such as long sample preparation, long analysis times, and high costs. In recent years, a multitude of studies have focused on different modalities of this technology, both in the field of AAB, including free-label LC–MS/MS [16] and isobaric tags for relative and absolute quantification (iTRAQ) [19], and in other areas such health sciences [35,36].

This work, based on previous studies, aims to standardize and implement a novel methodology based on LC–MS/MS for the identification of thousands of proteins belonging to the members of the microbial communities responsible for vinegar production processes, especially AAB. This strategy should be reliable and reproducible, especially in studies working with vinegars obtained by submerged culture. The characterization of three acetification profiles for submerged culture using innovative raw materials (synthetic alcohol medium, fine wine, and craft beer) as acetification substrates, and working in a semicontinuous mode, was performed in three studies by applying this systematic approach [37,38,39]. In this way, the biodiversity and some basic aspects regarding the functionality and metabolic activity of the microorganisms involved in these bioprocesses were determined, allowing us to identify both the predominant taxa and others never before found in these environments.

## 2. Results

The LC–MS/MS-based methodology has enabled the study, in both qualitative and quantitative terms, of the protein profiles of microbiota members involved in three submerged acetification processes involving, first, a reference raw material (synthetic alcohol-based medium) and, second, two natural raw materials (fine wine and craft beer). The main findings obtained from the use of LC–MS/MS, as well as the development and standardization of this work strategy, are described herein; the full studies of Román-Camacho et al. may be consulted for additional content [37,38,39].

### 2.1. Qualitative Metaproteomics

**Figure 1:**
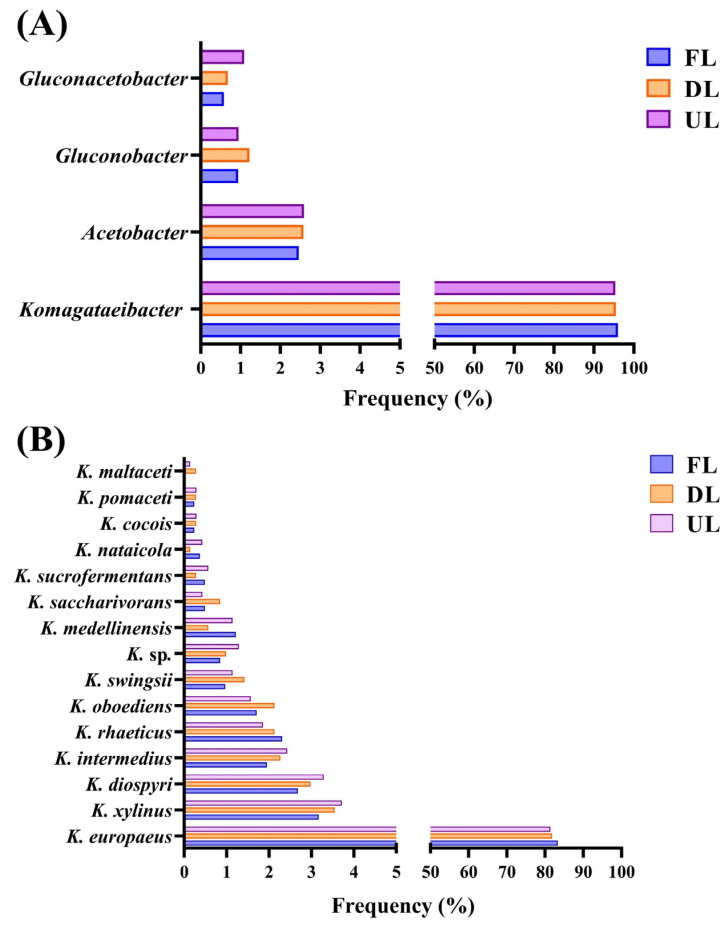
(**A**) Frequency of the four main (>1%) genera (*Acetobacter*, *Gluconacetobacter*, *Gluconobacter*, and *Komagataeibacter*) at each sampling moment (FL, end of fast loading; DL, end of discontinuous loading; UL, just before unloading). (**B**) Frequency of the species found in the genus *Komagataeibacter* at each sampling moment (FL, end of fast loading; DL, end of discontinuous loading; UL, just before unloading). Frequency (%) is represented as the number of proteins of each taxon out of the total proteins. Adapted by the authors from Román-Camacho et al. [37]. For a more detailed explanation of the operating mode and sampling times, see Section 4.3 and Section 4.4.

First, the study of the metaproteome was approached from a qualitative perspective, i.e., without considering the analysis of protein quantification values. The implementation of LC–MS/MS allowed the identification of thousands of proteins associated with different microorganisms for the microbiotas involved in the acetification profiles. This strategy also enabled the monitoring of the evolution of the composition and function of these metaproteomes throughout the acetification process in order to obtain a preliminary general overview. The LC–MS/MS analysis revealed that, in terms of verified proteins, acetic acid bacteria (AAB) were the most abundant microorganisms. Among the most abundant AAB genera, *Komagataeibacter* (95.6%), *Acetobacter* (2.5%), *Gluconacetobacter* (1.1%), and *Gluconobacter* (0.8%) were highlighted and represented 93.7% of the metaproteome in the synthetic alcohol medium profile; see Figure 1A. Despite the predominance of the aforementioned genera, between 25 and 30 genera of the family Acetobacteraceae, and more than 75 different species, were detected in the different acetification profiles identified. In addition to AAB, members of the acidophilic bacteria group, such as *Acidocella*, among others, were identified. Within the highly abundant genus *Komagataeibacter*, the species *K. europaeus* was highlighted in the three studies as providing the highest protein abundance, >70% in the different vinegar production processes; see Figure 1B. In short, the qualitative metaproteomic LC–MS/MS-based approach allowed us to clarify that the protein profiles were mainly composed of *K. europaeus*, closely related species, and a less-abundant fraction of numerous Acetobacteraceae members never before described in vinegar to date.

**Figure 2:**
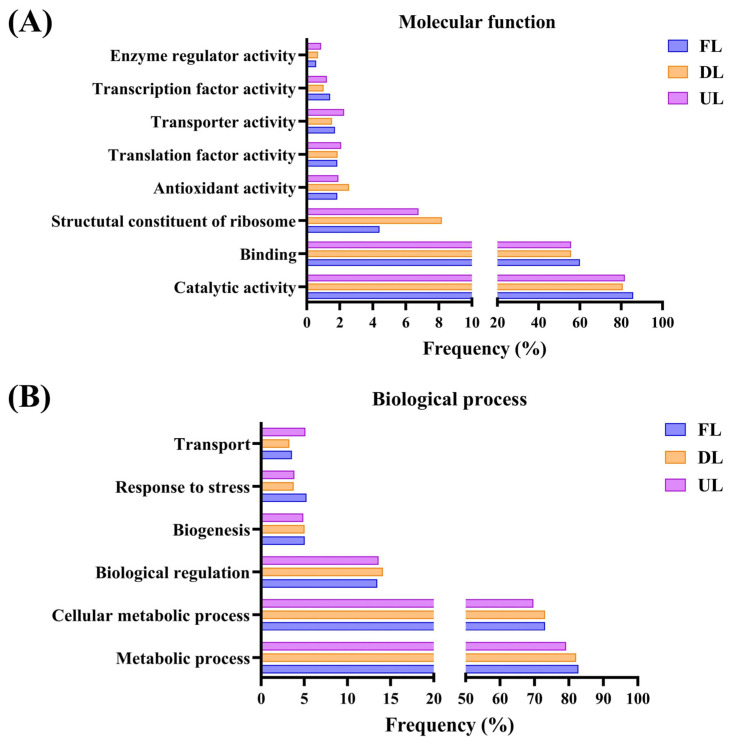
GO term enrichment analysis of the proteins found in the four main genera (>1%) at each sampling moment (FL, end of fast loading; DL, end of discontinuous loading; UL, just before unloading), represented as frequency (%) out of the number of proteins found in each GO term category: (**A**) molecular function and (**B**) biological process. Adapted by the authors from Román-Camacho et al. [37]. For a more detailed explanation of the operating mode and sampling times, see Section 4.3 and Section 4.4.

The qualitative metaproteomic LC–MS/MS-based approach also allowed us to perform functional studies to evaluate the behavior of the microbiota working in its natural environment. GO (gene ontology) term analyses of the predominant microbiota highlighted the important role of catalytic activity, organic cyclic compound binding, and metabolic biosynthetic processes throughout vinegar production; see Figure 2. Non-abundant species demonstrated their role in survival and stress response as these species attempted to establish a stable coexistence and functional relationship with the predominant species.

### 2.2. Quantitative Proteomics

**Figure 3:**
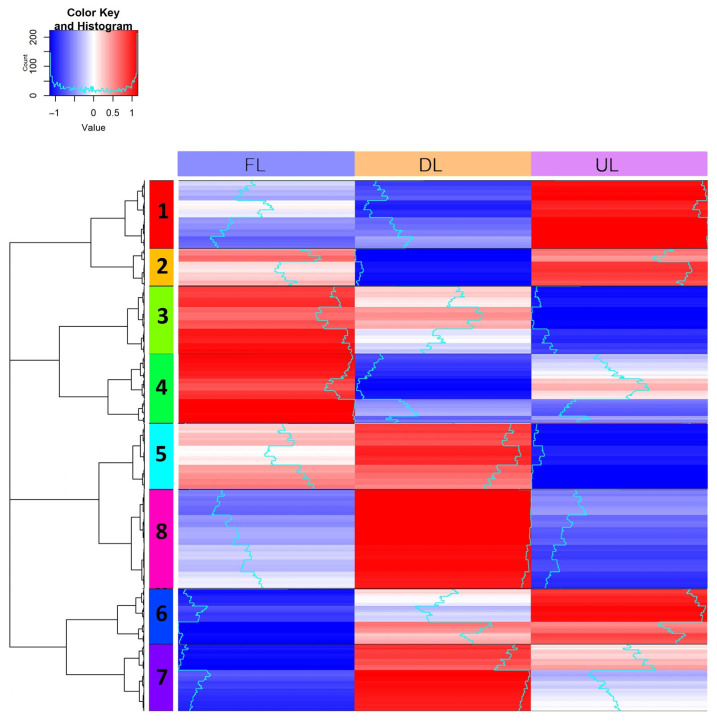
Heat map showing the hierarchical clustering analysis of the proteins of *K. europaeus* identified in ≥50% of the samples at each sampling moment (FL, end of fast loading; DL, end of discontinuous loading; UL, just before unloading). The numbers on each color correspond to each of the built clusters presenting more interactions than expected (PPI enrichment *p*-value < 0.05). Adapted by the authors from Román-Camacho et al. [38]. For a more detailed explanation of the operating mode and sampling times, see Section 4.3 and Section 4.4.

Next, the work strategy was focused on a more exhaustive study of protein quantification values, i.e., quantitative proteomics, to determine differences in protein activity in the microorganisms present throughout the acetification profiles. Enrichment LC–MS/MS and bioinformatic analyses of the proteome of the most representative species, *K. europaeus* (>70%), allowed us to obtain more comprehensive results; see Figure 3 and Figure S1 in Supplementary Materials. First, hierarchical clustering analysis allowed the establishment of protein groups with specific quantification patterns in the profiles (Figure 3). Then, the functional interactions of these protein groups were analyzed (Figure S1 in Supplementary Materials). The obtained results confirmed the importance of some metabolic processes, such as the biosynthesis of amino acids and proteins, as well as energy (ATP) generation-related pathways (TCA cycle and pentose phosphate pathway), whose proteins normally increased their quantification values in the first stages of acetification during the loading periods (FL and DL). Afterward, other proteins came into play, showing higher quantification values; these mainly included proteins related to acetic acid resistance in the final moments of acetification just before unloading (UL); see Figure S1 in Supplementary Materials. The quantitative approach of LC–MS/MS enabled new milestones to be achieved compared to the qualitative one. In this case, using *K. europaeus* as a study model, it was possible to more comprehensively describe the functional profile, including the evolution of the microbial activity and behavior in the acetification profile.

### 2.3. Proteomic Comparative Studies

LC–MS/MS also allowed for comparative metaproteomic and quantitative proteomic studies to be carried out, in particular, an analysis of the protein composition and function in the acetification profiles of fine wine and craft beer [39]. The implementation of a comparative study of the two profiles demonstrated that the use of different raw materials barely modified the protein profiles across the various acetification processes; however, it influenced a change in the metabolic behavior of the microorganisms at a quantitative level. This finding was supported by enrichment and subsequent quantitative proteomic analyses, including hierarchical clustering, a study of the quantitation patterns, and differential quantitation analysis (ANOVA and Tukey’s HSD; see Figure S2 in Supplementary Materials). In this way, protein groups involved in metabolic strategies were identified to be depending on both the acetification profile moment and the raw material; proteins involved in energy metabolism pathways (i.e., pentose phosphate pathway) were more abundant in the craft beer vinegar [green (B_EL) and yellow colors (B_UL) in Figure S2], while those related to biosynthesis processes (amino acids and nucleic acids) during the loading periods [green (B_EL) and purple colors (FW_EL) in Figure S2] and cellular detoxification at the membrane level in the final stages were predominant in the fine wine vinegar [blue color (FW_UL in Figure S2). TCA cycle proteins also displayed a critical role in the acetification profiles and were mainly associated with inner acetic acid metabolization. Therefore, in contrast to the individual studies (Section 2.1 and Section 2.2), the comparative study of the two acetification profiles via LC–MS/MS made it possible to establish the key metabolic strategies used by the predominant microbiota to take advantage of the resources offered by the raw materials at each moment of the profile.

## 3. Discussion

The complex ecosystems of fermented foods, which are mostly represented by AAB in the industrial production using acetic acid as the main component, constitute difficult environments to study due to the selective growth conditions of these microorganisms [5,40]. In this regard, a system for the identification and analysis of the members of these communities based on the study of the protein content via LC–MS/MS was fine-tuned in this work, specifically for the vinegar industry area.

One of the original methods used to study vinegar microbiota was two-dimensional electrophoresis (2DE), a technique that separates complex protein mixtures by employing isoelectric focusing (IEF) and molecular weight sorting with polyacrylamide gels (SDS-PAGE) [29]. In one of these first noteworthy studies, protein patterns in response to acetate stress in two acetate-resistant species of *Acetobacter* were determined [41]. Later, researchers investigated proteins linked to acetic acid response in *A. aceti* and their connection to the tricarboxylic acid (TCA) cycle as metabolization strategies [42,43]. However, although 2DE provided valuable insights into enhancing vinegar production, it faced reproducibility challenges between gels. To tackle this, researchers introduced differential gel electrophoresis (DIGE), which involves labeling two samples with distinct fluorescent dyes (Cy3-NHS and Cy5-NHS) before running them on the same gel [30]. Thus, by subsequently implementing 2D-DIGE, the identification of differentially quantified proteins in *A. pasteurianus* and the metaproteome of *Komagataeibacter* spp. involved in spirit vinegar production was facilitated [25,44]. Due to the labor-intensive nature of these protein identification methods, there was a push for new approaches to swiftly identify AAB and other related microorganisms. Matrix-assisted laser desorption ionization time-of-flight mass spectrometry (MALDI-TOF MS) is notable among these methods. This tool rapidly identifies considerable volumes of bacterial samples, generating a distinctive mass spectrum with peaks linked to high-abundance soluble proteins, resulting in unique protein profiles for bacterial groups and aiding differentiation at the genera, species, and strain levels [30,31]. MALDI-TOF MS has been tested for the quick and reliable identification of AAB on assays of elaboration of different varieties of vinegar and the detection of beer spoilage, among others [20,45,46]. Despite its usefulness, MALDI-TOF MS does not allow reliable quantification of protein samples and requires the isolation of microorganisms to obtain fresh material for their characterization [30,31]. Thus, in subsequent years, the fusion of mass spectrometry (MS) with liquid chromatography (LC) gave rise to LC–MS technology, known for its sensitivity, selectivity, and precision in separating and detecting analytes (peptides, proteins, other macromolecules, and metabolites) [32]. With the addition of a second mass analyzer, LC–MS/MS emerged as a potent tool for the rapid identification of thousands of analytes, as the proteins of a metaproteome, without prior electrophoresis-based separation [32,33]. This innovative approach, often considered within the shotgun technique, offers a broader dynamic range and enhanced protein coverage, making it valuable for recent studies of AAB by implementing different modalities (such as label-free LC–MS/MS or isobaric tags for relative and absolute quantitation (iTRAQ, TMT)) appropriate for exploring and characterizing complex metaproteomes, such as those present throughout vinegar making [16,19,33].

Thus, in our studies, by applying LC–MS/MS in a qualitative metaproteomic approach, abundant valid proteins belonging to a total of 25 to 30 different genera were identified. In the studies conducted by Andrés-Barrao et al. [25,46] using MALDI-TOF MS fingerprinting and 2D-DIGE, similar results were obtained regarding the most abundant taxa. First, 64 isolated strains of acetic acid bacteria (AAB) from vinegar and other similar media were characterized via MALDI-TOF MS fingerprinting as belonging to 22 different species from the genera *Acetobacter*, *Gluconacetobacter*, and *Gluconobacter* [46]. In high-acid fermentation of spirit vinegar, a homogeneous population of *Komagataeibacter* spp. was identified by 16S rRNA gene sequencing, and their differentially expressed proteins were analyzed by using 2D-DIGE and MALDI-TOF MS. *K. europaeus* was the most representative species in the metaproteome from our studies, which has been corroborated by other works using several molecular approaches such as PCR-based or gel-based approaches (16S–23S rRNA gene ITS regions) [22,31,47,48]. Although the composition of the most abundant fraction of the metaproteome was similar to that identified in other studies—such as the aforementioned studies which identified the predominance of *Komagataeibacter* spp., as *K. europaeus*, followed by other AAB genera such as *Acetobacter*, *Gluconacetobacter*, and *Gluconobacter*—to our knowledge, no previous works have detected so high a number of proteins and different genera, including members of the family Acetobacteraceae never before found in these media. Thanks to the wide range of coverage of LC–MS/MS, less-abundant protein fractions from other both AAB (e.g.,: *Asaia*, *Bombella*, *Swaminathania*, *Swingsia*, *Tanticharoenia*) and acidophilic genera (e.g.,: *Acidiphilium*, *Acidomonas*, *Oleomonas*, *Rhodovarius Roseomonas*) were identified, forming the most diverse vinegar metaproteome obtained to date [37,38]. Furthermore, LC–MS/MS solved many of the shortcomings of the other techniques, such as the lack of reproducibility between gels, excessive sample handling, and the need to obtain isolates from vinegar, allowing, in this case, sampling directly from vinegar, which offers greater reliability and reproducibility in the study of the evolution of the acetification process due to the high preservation of cell integrity and, therefore, of proteins [29,49,50,51].

After the identification of numerous species involved in the acetification processes, the proteome of *K. europaeus* was found to be the most representative in terms of protein composition and functionality. In this way, LC–MS/MS allowed us to perform an enriching study of this particular proteome, providing even more reliable proteins of *K. europaeus* than those of the initial metaproteome. LC–MS/MS enabled us to more comprehensively highlight and analyze key fractions or species within a complex metaproteome, which is a valuable strategy to consider [29,52]. Moreover, LC–MS/MS allowed for a quantitative proteomic study to be carried out, thus establishing protein clusters based on their quantitative patterns. Andrés-Barrao et al. [25] conducted differential protein expression studies, through ANOVA, of common spots (359) obtained by 2D-DIGE; in their study, most of these proteins were functionally related to stress response and the TCA cycle, among other diverse metabolic processes. Xia et al. [19] performed iTRAQ-based quantitative proteomic studies of *A. pasteurianus* Ab3 during vinegar fermentation; in their work, proteins involved in amino acid and fatty acid biosynthesis, as well as toxin–antitoxin systems, were differentially expressed, thus mobilizing the whole cellular system in response to high-acid stress. Yin et al. [16] applied LC–MS/MS for the study of the proteome of *A. pasteurianus* to understand its metabolism under the growing conditions for the L-aspartic acid and L-glutamate amino acids. The pentose phosphate pathway, the synthesis of nucleic acids, and unsaturated fatty acids were some of the highlighted processes. LC–MS was used to detect hydrolyzed gluten in commercial malt vinegar, including, more specifically, peptides derived from B-, D-, and γ-hordein from barley, as well as γ-gliadin, HMW-, and LMW-glutenins [34].

LC–MS/MS allowed us to not only characterize the metaproteome composition but also the function of the natural raw material acetification profiles and perform a detailed comparative study between them [39]. The quantitative analyses, through protein quantification values, determined different metabolic strategies; *K. europaeus*, as the most representative species, prevailed over the rest of the microbiota by leveraging the resources of each raw material according to three mechanisms: (1) metabolizing acetic acid via the TCA cycle to replenish cellular material losses; (2) utilizing excess glucose through the pentose phosphate pathway and glycolysis; and (3) triggering proton motive force-dependent membrane mechanisms to detoxify the cell at the end of acetification [38,53]. Although many of the identified key metabolic processes during vinegar production were consistent with those reported in other studies, LC–MS/MS not only allowed the description of some of them in more detail, as well as the enrichment of the *K. europaeus* proteome, but also provided more accurate quantification values than other proteomic techniques, covering a wider coverage of proteins along the acetification profile, thus allowing a comprehensive study of the dynamics of the process [31,32,34].

Other authors have used proteomic procedures [16,19,25,44,54], as well as those based on other omics technologies [20,27,55,56,57], to determine key metabolic processes of AAB, such as incomplete oxidation of ethanol to acetic acid, the metabolism of biomolecules (amino acids, nucleic acids, and fatty acids), energy generation related-pathways (TCA cycle and pentose phosphate pathway), and membrane-dependent high acidity stress response mechanisms, among others. LC–MS/MS allowed the determination of key metabolic processes and strategies for vinegar production and other biotechnological applications of interest to AAB [39]. The main advantages of the method developed based on LC–MS/MS over other molecular techniques were the ability to perform comparative analyses covering a wide range of protein coverage, allowing the detection of low abundant fractions, high precision in protein quantification, and suitability for in situ analysis of the fermentation process, without the need for isolation of microorganisms.

## 4. Materials and Methods

### 4.1. Raw Material

Three raw materials of different alcoholic origins were used as acetification media: synthetic alcohol medium, dry fine, and craft beer. In all cases, the ethanol concentration was adjusted to around 10% (*v*/*v*). Additional information on the composition of the raw materials can be found in the works of Román-Camacho et al. [37,38,39].

### 4.2. Starter Inoculum

A mixed culture collected from a functional industrial tank, namely, from the final stage of an active acetification process for producing alcohol vinegar, was used as the original inoculum for the three fermentation processes (UniCo Vinagres y Salsas, S.L.L., Doña Mencía, Cordoba, Spain). At the beginning of each profile, numerous previous cycles using each raw material are necessary for the calibration of the probes and the adaptation of the microorganisms.

### 4.3. Fermentation Conditions

**Figure 4:**
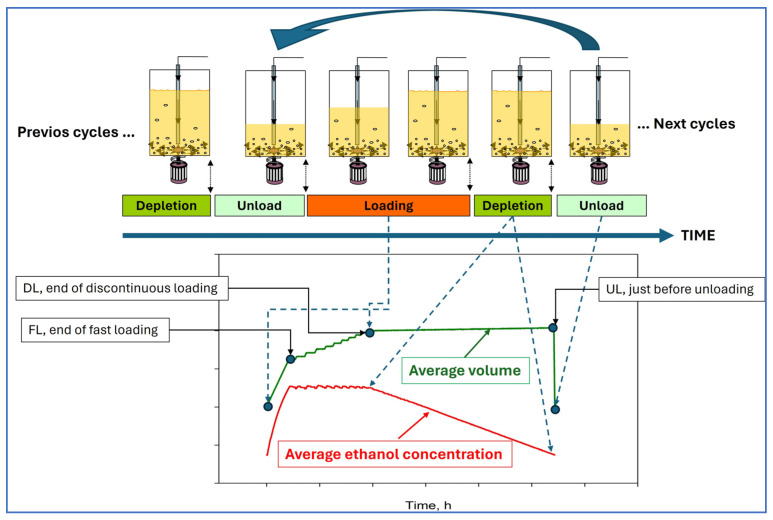
Diagram of the different phases of the semicontinuous acetification cycles and examples of mean profiles for ethanol concentration and volume of all the stable cycles.

The acetification profiles were conducted using a fully automated 8 L Frings reactor (Heinrich Frings GmbH & Co., KG, Bonn, Germany). The reactor was operated in a semicontinuous mode, which involved the gradual reduction in ethanol in the medium to a specified concentration range (1.0%, *v*/*v*). Once this ethanol concentration was achieved, approximately 50% of the reactor content was unloaded (4 L). Subsequently, the reactor was refilled until reaching the final working volume (8 L) while ensuring that the preset ethanol concentration (5%, *v*/*v*) was not exceeded; in practice, in order to not exceed the 5% ethanol concentration, the loading necessary to reach 8 L involved two phases: a first, fast phase that added a volume of the fresh medium until this ethanol concentration was reached, and, from that moment on, a second, slow loading phase that added the necessary volume in a discontinuous manner in order not to exceed 5% ethanol until the total volume of 8 L was reached; see Figure 4. A constant temperature of 31 °C, a loading rate of 1.3 ± 0.1 L/h, and an airflow rate of 7.5 L (h L medium)^−1^ were established in all cases.

### 4.4. Sampling

First, a reactivation and inoculum adaptation phase were conducted, including several previous acetification cycles, to achieve a stable and repetitive semicontinuous system. Once the acetification profile was defined, numerous stable cycles elapsed where samples were collected at several key moments along the profile. For the acetification profile of the synthetic alcohol medium, sampling was carried out in 28 stable cycles and three cycle phases: at the end of fast loading (FL), at the end of discontinuous loading (DL), and just before unloading (UL). For the acetification profiles of the natural raw materials [dry fine wine (FW) and craft beer (B)], because of a faster ethanol disappearance rate, there was only a final loading phase (EL) in addition to the state immediately before the unloading state (UL); a total of 15 stable cycles were conducted for each of these profiles. In all cases, three or four biological replicates were obtained at each sampling time.

### 4.5. Cellular Collection

Each vinegar sample was obtained directly from the reactor, divided into 6–8 fractions containing 300–400 mL, and then transferred to Falcon^TM^ 50 mL High Clarity Conical Centrifuge Tubes (Thermo Fisher Scientific, Waltham, MA, USA) placed on ice water. The cells from the vinegar samples were harvested through centrifugation at 4 °C and 4600× *g* for 10 min (Hettich, Rotina 38R, Westphalia, Germany). The resulting cell pellets were subjected to two rounds of washing and homogenization with 1 mL of cold sterile distilled water or PBS buffer (phosphate-buffered saline, pH 7.2–7.4). Simultaneously, the cells were collected in Eppendorf tubes via centrifugation (twice) at 4 °C and 16,900× *g* for 1 min. These cell pellets were stored at −80 °C until proteomic analysis.

### 4.6. Protein Extraction

The cell pellets obtained in the preceding steps were resuspended in 600 μL of an extraction buffer composed of 100 mM Tris-HCl buffer (pH 8.0), 2 mM DTT, 1 mM EDTA, and protease inhibitor cocktail tablets. For cell lysis, an equivalent volume of glass beads to that of the total volume in the Eppendorf tube was added. The cell disruption process involved vortexing (10 cycles of 1 min of vortexing followed by 1 min on ice) and subsequent sonication using an ultrasonic cleaning bath (J.P. SELECTA^®^, Ultrasons, Barcelona, Spain). Then, both the glass beads and cell debris were removed through a double centrifugation process, first at 7000× *g* for 10 min at 4 °C and then at 16,000× *g* for 20 min at 4 °C.

**Figure 5:**
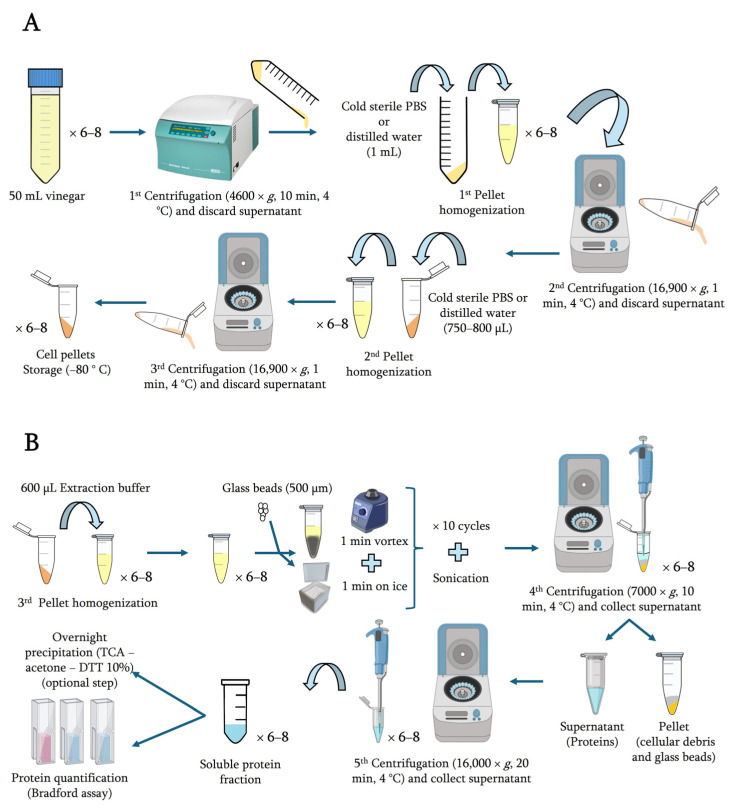
Workflow for the processing of vinegar samples for protein extraction and quantification targeted to LC–MS/MS analysis. (**A**) Cell collection from vinegar samples; (**B**) cell lysis, extraction of soluble protein fraction, and quantification. Created with BioRender.com.

An optional phase to optimize protein recovery involves precipitation by incubating the protein supernatant overnight at −20 °C following the addition of 4 volumes of ice-cold TCA-acetone-DTT solution (10% *w*/*v*). However, if an adequate cellular and, therefore, protein content was estimated, this step is usually not necessary. After incubation, the samples were centrifuged at 16,000× *g* for 40 min at 4 °C, resulting in protein pellets. These protein pellets were then subjected to two washes with 500 μL of ice-cold acetone-DTT solution (0.07% *w*/*v*), followed by storage at −20 °C for 30 min and subsequent centrifugation at 15,000× *g* for 15 min at 4 °C. The resulting protein pellets were further processed via vacuum drying in a SpeedVac^TM^ concentrator (Thermo Fisher Scientific, Waltham, MA, USA) to remove acetone residues. Subsequently, they were resuspended in 600 μL of a solubilization buffer consisting of 8 M urea, 2% CHAPS, and 20 mM DTT. The solubilization phase included several shaking cycles of 30–45 min at 1200 rpm and 4 °C. The solubilized samples were then centrifuged at 16,000× *g* for 15 min at 4 °C; the supernatant contained a soluble protein fraction whose concentration was estimated according to Bradford assays [33]. The protein extraction method is detailed in Figure 5.

### 4.7. LC–MS/MS Analysis

For the LC–MS/MS analysis, ≥50 μg of proteins from each sample were processed at the Research Support Central Service (SCAI), University of Córdoba, Spain.

The samples were cleared by means of SDS-PAGE by concentrating the extracts in an approximately 1 cm band of the resolving gel. Then, they were stained with Coomassie blue and trimmed for protein digestion. After the destaining, washing, and reduction/alkylation steps in 25 mM of ammonium bicarbonate, the samples were digested with trypsin at 12.5 ng/µL at 37 °C overnight. The nano-LC analyses were conducted using a Dionex Ultimate 3000 Nano UHPLC (Thermo Fisher Scientific, MA, USA) system equipped with an Acclaim Pepmap C18 separation column (500 mm × 0.075 mm). The peptide mixtures were initially trapped using an Acclaim Pepmap C18 pre-column (5 mm × 0.3 mm) at a flow rate of 5 μL/min for 5 min, employing a solution of 2% acetonitrile and 0.05% trifluoroacetic acid. The peptide separation was performed at 40 °C for all runs, employing a 60 min gradient, ranging from 4% to 90% acetonitrile with 0.1% formic acid, and a flow rate of 300 nL/min.

The analysis of eluting peptide cations was achieved using a mass spectrometer, specifically, the Orbitrap Fusion (Thermo Fisher Scientific, Waltham, MA, USA), which features a nanoelectrospray ionization interface. Survey scans of peptide precursors within the 400 to 1500 *m*/*z* range were carried out at a resolution of 120K (at 200 *m*/*z*) with a target ion count of 4 × 10^5^. The tandem MS analysis involved isolating ions with a 1.2 Th window at the quadrupole, performing CID fragmentation with a normalized collision energy of 35, and conducting rapid scan MS in the ion trap. The AGC ion count target was set at 2 × 10^3^ with a maximum injection time of 50 ms. For MS/MS, only precursors with charge states ranging from 2 to 5 were selected. The dynamic exclusion duration was set to 15 s, with a 10 ppm tolerance around the selected precursor and its isotopes, and monoisotopic precursor selection was enabled. The instrument operated in top-speed mode with 3-s cycles.

The raw data from the mass spectrometry were processed using Proteome Discoverer (version 2.1.0.81, Thermo Fisher Scientific, MA, USA). MS/MS spectra were analyzed using the SEQUEST search engine against UniProt-specific databases. Peptides resulting from tryptic digestion were examined with the following parameters: up to one missed cleavage, cysteine carbamidomethylation as a fixed modification, and methionine oxidation as a variable modification. A precursor mass tolerance of 10 ppm was applied, and we searched for ion products with tolerances of 0.1 Da. Validation of peptide spectral matches (PSM) was conducted at a 1% false discovery rate (FDR) using a percolator based on *q*-values. Peptide quantification was performed by calculating the precursor ion areas using a precursor ion area detector and through normalization using the total peptide amount mode of Proteome Discoverer. The parsimony principle, filtered to achieve a 1% FDR, was used to obtain protein groups.

### 4.8. Raw Data Analysis

The identified proteins were filtered to only retain those with high protein FDR confidence (<0.01), removing those with a score < 2 and a peptide number ≤ 2. Then, proteins identified in ≥50% of the replicates in at least one sampling time were retained for further analyses. Excluded proteins were defined as those identified in ≥50% of the replicates of a given sample and not detected in any replicate of the other sample in the context of pairwise comparison and were excluded from the total count. GO term analysis, by means of UniProt and gene ontology (GO) annotation tools, is usually employed to elucidate functional aspects of proteins. For quantitative proteomic studies, LC–MS/MS enrichment analyses were carried out with an emphasis on the monitoring of quantitative variations. Protein quantification values were corrected by global sample intensity; thus, each quantification value was divided by the overall intensity of the sample and then multiplied by the mean intensity value derived from all samples. Those proteins present in ≥50% of the samples at a given sampling time were selected and subjected to an intersection analysis using the “UpSetR” R library. For hierarchical clustering and heat map analysis, proteins identified in ≥50% of the samples at each sampling time were analyzed. The mean quantification values were first scaled and centered through z-score transformation. Subsequently, Pearson correlation with the “complete” method was applied using the “hclust” function in the stats R package. One-way ANOVA followed by HSD Tukey’s testing was computed using R functions “lm” and “anova,” and *q*-value was employed for multiple testing correction of *p*-values. Moreover, the functional role of protein clusters was explored by constructing protein–protein interaction network maps (INMs) utilizing the STRING database (https://string-db.org/, accessed on 4 January 2021). Protein annotations sourced from the UniProt and KEGG databases were considered.

## 5. Conclusions

In this work, an LC–MS/MS-based method was developed which, through the application of different metaproteomic approaches, enabled the characterization of the microbiota present during the evolution of three submerged vinegar production profiles. This work strategy was tested in three studies and should be reproducible and applicable for the identification and characterization of complex microbial communities present in vinegar and related industries. LC–MS/MS enabled the determination of both the predominant microbial groups and others never found in vinegar, as well as the determination of the functional profile of the microbiota. These findings may be crucial for collecting strains from the most abundant species to obtain improved starter cultures, as well as for determining marker metabolic pathways and processes for the vinegar industry. Subsequent steps in the advancement of this research should target the use of LC–MS/MS in conjunction with other omics tools for integrated “multi-omics” profiling. Furthermore, data-independent acquisition (DIA), together with software development tools for data analysis, has recently emerged as the most interesting tool for quantitative and deep proteomics; therefore, it may be useful if implemented in the vinegar field to address challenges faced in the design of efficient methodologies for the characterization of AAB and other vinegar microorganisms beyond the Acetobacteraceae family [58].

## Data Availability

Mass spectrometry proteomics data from Román-Camacho et al. [39] have been deposited to the ProteomeXchange Consortium via the PRIDE partner repository with the dataset identifier PXD031147.

## Supplementary Materials

The following supporting information can be downloaded at: https://www.mdpi.com/article/10.3390/molecules29112548/s1, Figure S1. Protein–protein interaction network maps (INMs) performed in *K. europaeus* of each cluster using STRING v11.0; those with a PPI enrichment *p*-value < 0.05 are shown. Proteins are shown as nodes, and interactions between them are represented by edges whose thickness indicates the strength of the interaction. Nodes with the same color represent specific protein groups according to MCL (Markov cluster algorithm); the most relevant were circled. (A) INM 3 (Cluster 3): red (TCA cycle, FDR = 2.1 × 10^−10^), slate blue (aminoacyl-tRNA biosynthesis, FDR = 1.4 × 10^−6^); (B) INM 4 (Cluster 4): red (biosynthesis of amino acids, FDR = 4.0 × 10^−2^), green (aminoacyl-tRNA biosynthesis, FDR = 4.0 × 10^−2^), cyan (glutathione metabolism, FDR = 9.7 × 10^−3^); (C) INM 5 (Cluster 5): sky blue (aminoacyl-tRNA biosynthesis, FDR = 9.5 × 10^−5^), red (ATP-binding, FDR = 1.0 × 10^−8^ and biosynthesis of amino acids, FDR = 1.80 × 10^−4^), orchid (peptidoglycan biosynthesis, FDR = 1.5 × 10^−2^); (D) INM 6 (Cluster 6): red (ribosome, FDR = 1.6 × 10^−4^); (E) INM 7 (Cluster 7): red (ribosome, FDR = 9.9 × 10^−9^); (F) INM 8 (Cluster 8): red (ribosome, FDR = 1.5 × 10^−5^), pink, orchid, and slate blue (purine and pyrimidine metabolism, FDR = 3.2 × 10^−2^). Figure S2. Radar chart including those proteins with the strongest significative differences based on HSD Tukey’s test corrected by multiple testing (*q*-value < 0.01) and log_2_ fold change (in absolute value) > 2. The quantification area of each protein in each profile (FW, fine wine; B, beer) and sampling time (EL, end of loading; UL, just before unloading) is shown.
