# Supplementary figures and images for: Implementation of a Novel Method for Processing Proteins from Acetic Acid Bacteria via Liquid Chromatography Coupled with Tandem Mass Spectrometry
Source: Molecules. 2024 May 29;29(11):2548. doi: 10.3390/molecules29112548 (PMC11173641; doi:10.3390/molecules29112548)

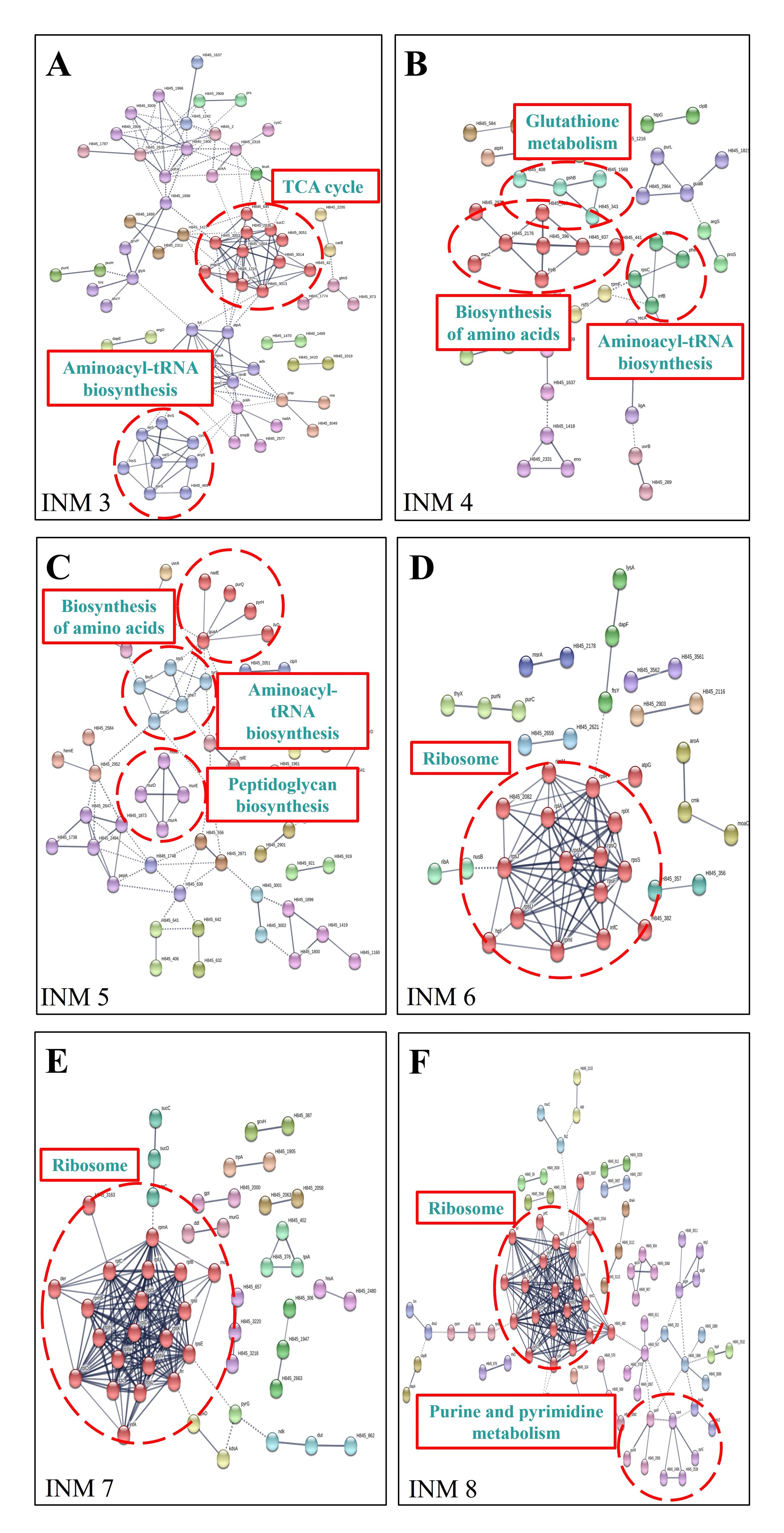

Supplement: Supplementary file 1 [file molecules-29-02548-s001.zip › Figure S1.jpg]

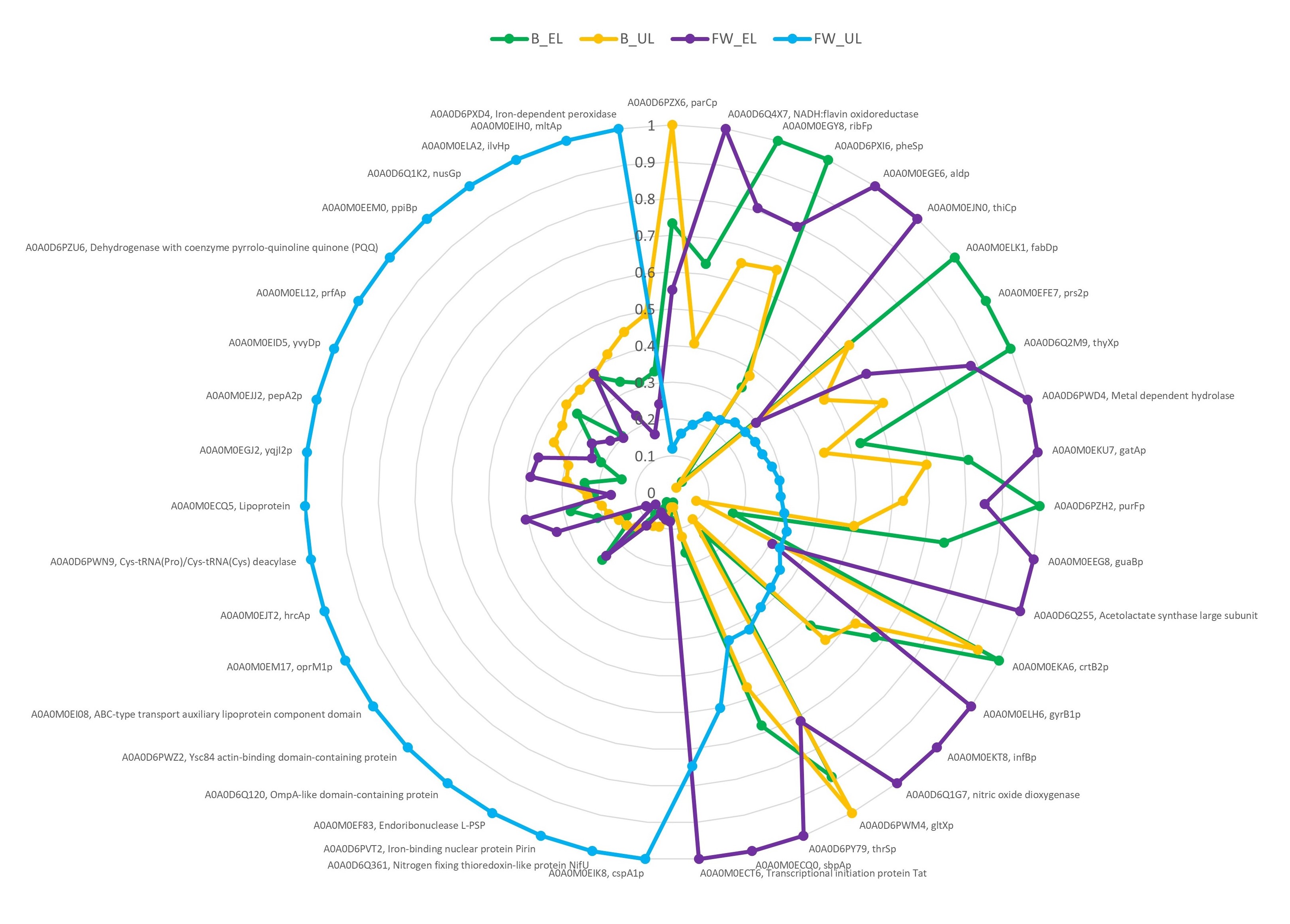

Supplement: Supplementary file 1 [file molecules-29-02548-s001.zip › Figure S2.jpg]
